# Intra-Operative Tumour Detection and Staging in Pancreatic Cancer Surgery: An Integrative Review of Current Standards and Future Directions

**DOI:** 10.3390/cancers16223803

**Published:** 2024-11-12

**Authors:** Ahmed Kotb, Zaynab Hafeji, Fadel Jesry, Nicole Lintern, Samir Pathak, Andrew M. Smith, Kishan R. D. Lutchman, Daniel M. de Bruin, Rob Hurks, Michal Heger, Yazan S. Khaled

**Affiliations:** 1 Leeds Institute of Medical Research, University of Leeds, Leeds LS2 9JT, UK; 2 The Pancreato-Biliary Unit, St James’s University Teaching Hospital, Leeds LS9 7TF, UK; 3 Department of Surgery, Amsterdam UMC, Location AMC, 1105 AZ Amsterdam, The Netherlands; 4 Department of Biomedical Engineering and Physics, Amsterdam UMC, Location AMC, 1105 AZ Amsterdam, The Netherlands; 5 Department of Radiology and Nuclear Medicine, Amsterdam University Medical Center, 1105 AZ Amsterdam, The Netherlands; 6 Jiaxing Key Laboratory for Photonanomedicine and Experimental Therapeutics, Department of Pharmaceutics, College of Medicine, Jiaxing University, Jiaxing 314001, China

**Keywords:** pancreatic cancer, imaging, intra-operative staging, fluorescence, tumour, lymph nodes

## Abstract

Background: Surgical resection for pancreatic ductal adenocarcinoma (PDAC) entails the excision of the primary tumour and regional lymphadenectomy. This traditional strategy is challenged by the high rate of early recurrence, suggesting inadequate disease staging. Novel methods of intra-operative staging are needed to allow surgical resection to be tailored to the disease’s biology. Methods: A search of published articles on the PubMed and Embase databases was performed using the terms ‘pancreas’ OR ‘pancreatic’ AND ‘intra-operative staging/detection’ OR ‘guided surgery’. Articles published between January 2000 and June 2023 were included. Technologies that offered intra-operative staging and tailored treatment were curated and summarised in the following integrative review. Results: lymph node (LN) mapping and radioimmunoguided surgery have shown promising results but lacked practicality to facilitate real-time intra-operative staging for PDAC. Fluorescence-guided surgery (FGS) offers high contrast and sensitivity, enabling the identification of cancerous tissue and positive LNs with improved precision following intravenous administration of a fluorescent agent. The unique properties of optical coherence tomography and ultrasound elastography lend themselves to be platforms for virtual biopsy intra-operatively. Conclusions: Accurate intra-operative staging of PDAC, localisation of metastatic LNs, and identification of extra-pancreatic disease remain clinically unmet needs under current detection methods and staging standards. Tumour-specific FGS combined with other diagnostic and therapeutic modalities could improve tumour detection and staging in patients with PDAC.

## Simple Summary

Pancreatic cancer can be cured by surgical resection to clear margins, but this is difficult to achieve. The current methods to detect cancer spread during surgery are not very effective and many patients experience cancer returning soon after surgery. This integrative review analyses new technologies that could aid surgeons in detecting cancer more accurately during surgery. The aim is to identify methods that allow surgeons to distinguish cancerous tissues and subclinical deposits more precisely and reduce the risk of cancer recurrence. Fluorescence-guided surgery, where a light-emitting dye illuminates cancer cells, and other cancer localisation techniques are emerging as promising tools to aid oncological clearance during surgery. Accurate identification of cancer spread during surgery remains a clinical need and could change how surgeries for pancreatic cancer are conducted to prevent post-resection recurrence and improve survival.

## 1. Introduction

Pancreatic ductal adenocarcinoma (PDAC) is predicted to be the second most common cause of cancer deaths by 2030, with little hope of improvement in the current 5-year survival rate [1]. Advancements in adjuvant and neoadjuvant chemotherapy have produced marginal benefits, with the 5-year overall survival rate increasing from 6% to 9% between 2014 and 2018 [2]. Complete surgical resection remains the only curative option for PDAC but is seldom achieved, with positive resection margins (R1) reported in up to 70% of cases [3,4,5]. When complete surgical resection is performed, 5-year survival rates of approximately 40% are reported [6,7]. Up to 26% of patients are found to have distant metastases during surgical exploration, which unsurprisingly is associated with a significantly decreased 5-year survival rate (<20%) [8,9,10]. Around 85% of patients will develop recurrence after surgical resection [11,12]. Some patients never receive adjuvant treatment and only 55–75% of patients who initiate adjuvant therapy complete the course [13].

Pancreatic cancer resection aims to remove the cancer while keeping the risk of morbidity and mortality low. Some authors suggest that local recurrence after surgical resection for PDAC is caused by incomplete lymph node (LN) clearance. However, extended lymphadenectomy (EL) does not improve survival [14]. A meta-analysis of 1909 patients comparing pancreatic cancer surgery with either a standard lymphadenectomy (SL) or an EL found no difference in overall survival. However, EL surgery did improve LN yield and the ability to achieve complete resection and facilitated survival prediction [15,16]. Accordingly, current evidence does not support EL over traditional SL in PDAC treatment [17].

The lack of real-time intra-operative disease identification to allow a complete resection, detection of involved LNs, and eradication of micrometastases is the leading cause of incomplete tumour removal and subsequent regrowth [18]. Current pre-operative imaging modalities lack the sensitivity to detect small lesions [19,20]. There is an unmet clinical need to enhance intra-operative cancer detection and improve the rates of complete cancer resection in PDAC.

A solution would be to personalise surgery by stratifying the resection’s radicality to the stage. Given the limits of the available imaging modalities, several researchers are concentrating on cutting-edge methods for intra-operative cancer detection. This provides various benefits and the option of minimising resection while achieving negative resection margins [21]. Firstly, intra-operative cancer detection could help identify patients with LN micro-metastases undetected by standard imaging techniques [22]. Secondly, it could eliminate the difficulties faced when distinguishing tumour tissue associated with the increased adoption of neoadjuvant chemotherapy. Thirdly, it would be helpful in specific clinical situations, such as staging laparoscopy, to identify peritoneal metastases, liver metastases, and extra-pancreatic LNs.

This review examines the limits of current imaging techniques in PDAC staging and addresses novel and emerging technologies for intra-operative tumour localisation. Additionally, the benefits and downsides of these techniques are evaluated, and their possible translation to clinical practice is discussed.

## 2. Imaging Modalities for PDAC Diagnosis and Staging

Accurate pre-operative staging/imaging is crucial in diagnosing PDAC [23]. To date, computed tomography (CT), magnetic resonance imaging (MRI), and positron emission tomography (PET) are employed to predict disease stage and resectability. Endoscopic ultrasound (EUS) can supplement existing imaging modalities by providing valuable staging information and enabling minimally invasive histological diagnosis via fine-needle aspiration or tissue biopsy.

### 2.1. Computed Tomography (CT)

Multidetector CT (MDCT) is the first-line imaging modality for assessing suspected PDAC due to its exceptional spatial resolution, cost-effectiveness, and global availability [24]. The sensitivity of CT imaging in detecting PDAC has significantly advanced over time, falling within the 89–97% range. Nonetheless, the detection sensitivity decreases to 67% for smaller lesions that measure less than 1.5 cm [25,26], which equates to 6–10% of the total length of an adult pancreas. A meta-analysis, which juxtaposed various imaging techniques used in the detection of PDAC, found that the sensitivity and specificity of CT imaging, at 89% and 90%, respectively, were on par with those observed with MRI [27].

On a CT scan, a PDAC classically appears hypodense in the arterial phase scans with indistinct boundaries [15]. Various changes that may raise suspicions of PDAC range from least to most specific and include dilation of the pancreatic duct (sensitivity 50%, specificity 78%), hypo-attenuation (sensitivity 75%, specificity 84%) [21], atrophy in the distal part of the pancreas (sensitivity 45%, specificity 96%), irregularities in the contour of the pancreas (sensitivity 15%, specificity 92%), and enlargement of the common bile duct (sensitivity 5%, specificity 92%) [28].

The ability of computed tomography (CT) to accurately differentiate malignant regional LNs in pancreatic cancer is significantly limited. Currently, LNs are classified as “suspicious” based solely on a size threshold of 1 cm. The spatial resolution of standard CT scanners ranges between 5 and 10 mm. However, this approach has inherent limitations, as large nodes can be benign and reactive, while normal-sized nodes can still harbour malignancy. In a retrospective study conducted by Raman et al. of 136 patients, the sensitivity of CT scans for detecting LNM was 22% [29]. A further study on 80 patients assessed the diagnostic accuracy of CTs in detecting LNM. They found a sensitivity, specificity, positive predicted value (PPV), and negative predicted value (NPV) of 89.7%, 82.4%, 74.3%, and 93.3%, respectively. However, this high sensitivity rate was only observed in LNM with a short axis diameter of ≥5 mm per LN [30].

CT scans provide wide accessibility and rapid assessment of possible PDAC diagnosis. However, additional imaging may be required to differentiate between benign or malignant conditions, detect iso-attenuation in primary tumours, or distinguish lesions smaller than 1 cm [31,32].

### 2.2. Magnetic Resonance Imaging (MRI) and Magnetic Resonance Cholangiopancreatography (MRCP)

MRI is favoured in many circumstances, including detecting PDAC tumours less than 2 cm and iso-attenuating lesions [29]. A meta-analysis of 68 papers reported that MRI demonstrated a sensitivity and specificity of 86% and 79%, respectively, in detecting PDAC [33]. Magnetic resonance cholangiopancreatography (MRCP) has proved to be an effective method for closely examining the biliary and pancreatic ducts in diagnosing PDAC, exhibiting a specificity and sensitivity of 80 and 90%, respectively [34]. MRIs, however, are hampered by artefacts that might conceal tumour architecture, are contraindicated in specific populations, and have low contrast resolution in the pancreas [35]. When evaluating cystic lesions in the pancreas, MRI is favoured over CT because it can more accurately detect enlarged LNs and distant metastases [36].

Lee et al. (2019) prospectively investigated the diagnostic potential of LNs using MRI features in pancreaticobiliary cancers (n = 36), including maximum diameters in the short and long axes, shape, signal intensities on T1- and T2-weighted imaging, the pattern of enhancement, and apparent diffusion coefficient (ADC) on diffusion-weighted MR images of LNs with measurable diameters of ≥ 5 mm in short-axis diameter. On multivariate analysis, the authors reported impressive sensitivity, specificity, overall accuracy, and positive predictive values, and NPVs of 89.7%, 82.4%, 85.0%, 74.3%, and 93.3%, respectively. However, the study included only a few patients with PDAC (n = 5). Interestingly, most small metastatic LNs were found in patients with pancreatic cancer in the peripancreatic or peritumoral area. In these cancers, detectable nodes were primarily located in LN stations 8 and 12, whereas peripancreatic node detection was poor. This observation may indicate that the pancreatic cancers often had small metastatic nodes in the peritumoral/peripancreatic area, consistent with the LN downstaging rate to cN0 being approximately 38%, with an NPV of 49.8% for LN metastasis [30]. In sharp contrast, in a recent retrospective study of 75 patients who underwent pancreaticoduodenectomy and lymphadenectomy for PDAC, none of the qualitative variables evaluated on MRI were associated with pathological nodes. Others reported improved diagnosis with a sensitivity of 72% of positive LNs in PDAC using MRI radiomics [37]. A further meta-analysis also concluded the superiority of MRI over CT scans in detecting liver metastases (sensitivity of 83% vs. 45% and specificity of 96% vs. 94%) [38]. One of the challenges in assessing the detection rate of LNM on MRI is the need for more node-by-node correlations between pre-operative MRI and histopathology samples. In addition, positioning a region of interest accurately to measure can be challenging due to some LNs’ diminutive size [39]. Given the prognostic implications of pathological LNs in these patients, improved diagnostic techniques are needed.

### 2.3. Positron Emission Tomography (PET)

The only molecular imaging method currently used for PDAC is PET. PET/CT, with its ability to depict high metabolic activity using an 18F-FDG tracer, can accurately determine the metabolic capacity and anatomical position of pancreatic tumour cells. It is advantageous in early diagnosis, accurate staging, and predicting patient survival. A clinical study conducted on 467 patients showed that 18F-FDG PET/CT was more accurate than enhanced CT or enhanced MRI in diagnosing pancreatic lesions, with sensitivity and specificity rates of 91.9% and 96.3%, respectively [40]. Accordingly, 18F-FDG PET/CT is a valuable tool for diagnosing pancreatic lesions. However, a joint application rather than a sole diagnostic method would strengthen diagnostic ability in PDAC [41].

The role of PET/CT in locoregional nodal staging has remained ambiguous, with no definitive benefits established to date. A significant number of recent studies have reported limited sensitivities for nodal status, ranging between 70% and 80% [42,43]. This suggests that the effectiveness of PET/CT in accurately determining nodal status is still questionable. Despite these findings, it is essential to note that the sensitivity of PET/CT, although moderate, was still higher than that of multidetector CT. The prospective multicentre PET-PANC study aimed to assess the incremental diagnostic accuracy and impact of PET/CT compared to multidetector CT in patients suspected of having pancreatic cancer [44]. The study, which included 550 patients, found that PET/CT correctly staged significantly more patients with stage IIb disease (pN+) than multidetector CT (*P* = 0.002). However, this resulted in only a moderate sensitivity of 38% for PET/CT, compared to 22% for multidetector CT. This suggests that PET/CT may potentially benefit staging pancreatic cancer, particularly in patients with stage IIb disease. However, more research is needed to confirm these findings and to further explore the potential role of PET/CT in locoregional nodal staging.

The concern with employing 18F-FDG uptake is that glucose metabolism is not specific to malignant processes, and physiologic uptake can be observed in both standard and inflamed tissues, which may lead to false-positive findings where pancreatitis and PDAC seem alike [45]. According to reports, non-tumour tissue can absorb up to 40% of FDG post-infusion [46]. Although FDG PET/CT is not standardly implemented for the purposes discussed here, it is undoubtedly evident from the examination of the literature that clinical practice should substantially incorporate FDG PET/CT. FDG PET/CT demonstrated solid efficacy in managing patients with PDAC, particularly when considering initial staging and treatment planning [47]. Indeed, more randomised trials are required to offer stronger evidence of FDG PET/CT’s instrumental position in the staging of patients with PDAC.

### 2.4. Endoscopic Ultrasonography (EUS)

The accuracy of endoscopic ultrasound (EUS) in tumour-node-metastasis (TNM) staging and determining vascular invasion is highly variable across studies [8]. Some studies indicate that EUS outperforms other imaging modalities, while others demonstrate comparable or even inferior results for EUS [9]. Several factors, including the experience of the endosonographer and their knowledge of prior diagnostic imaging studies, may influence the staging accuracy of EUS. Both radial and linear array EUS instruments exhibit similar performance, although the latter allows for fine needle aspiration (FNA) [48] and fine needle biopsy [48].

EUS combines the advantages of endoscopy, which enables direct visualisation of the gastrointestinal lumen, with the benefits of ultrasound imaging, which provides detailed structural information [49]. EUS also allows for tissue collection and cytological analysis, both of which can offer a conclusive diagnosis. Referring to a recently published meta-analysis, the sensitivity and specificity of EUS-FNA for the diagnosis of pancreatic adenocarcinoma are 85% and 98%, respectively [50].

EUS has grown to be a useful diagnostic tool for PDAC. When evaluating solid pancreatic tumours, EUS has a higher sensitivity (98%) than CT (86%) for lesions under 2 cm [51]. More importantly, isolated lesions as small as 2 mm can be found with EUS [52]. In comparison to CT (69%) and MRI (82%), a retrospective study of 140 patients found that EUS had remarkably higher accuracy (91%) in the diagnosis of PDAC lesions measuring less than 20 mm [53]. A meta-analysis of 29 studies indicated that EUS has high specificity (90%) but only moderate sensitivity (73%) in detecting vascular invasion [54]. However, utilising EUS to assess solid pancreatic lesions has potential drawbacks. These include operator dependency, causing varying sensitivities ranging from 57% to 81% [55].

The range of EUS accuracy for PDAC LNM staging is 41–86% [56,57]. When diagnosing LNM in PDAC, EUS alone often has a sensitivity of less than 65% but a specificity of more than 70% [57,58]. Alongside its invasiveness, detecting extra-abdominal metastases is another limitation. As a result, CT or MRI must be used to complete the PDAC staging.

## 3. Intra-Operative Staging Modalities

Intra-operative staging and diagnosis of PDAC vary significantly due to the variety of methods used, and there is currently no gold standard approach. The goal is to accomplish tumour-free surgical resection (R0) while utilising a minimally invasive technique, which presently is challenging.

### 3.1. Established Techniques

#### 3.1.1. Frozen Sections

Frozen sections of surgical margins are employed by some surgeons to improve the likelihood of achieving an R0 resection. Frozen section examination by a pathologist and intra-operative ultrasound are the only techniques a pancreatic surgeon can use to ensure an R0 resection [59].

The disadvantage of this margin control technique in confirming an R0 resection is the impossibility of sampling the entire tumour surgical bed. The process is operator-dependent and increases the risk of false-negative results [60]. According to studies, the accuracy of frozen sections in diagnosing PDAC is limited, with sensitivities ranging from 33 to 93% and specificities ranging from 73 to 100% [61].

However, due to the lengthy process and limited sampling of the surgical bed in frozen section analysis, it has been documented that false-negative results can reach as high as 75% [62]. This comparatively high rate of false negatives leads us to question whether a clinical judgement may be more reliable than a negative frozen section appraisal. A multi-centre analysis involving 1,399 patients found that supplemental resection based on frozen section guidance yielded no increase in overall survival [63]. Moreover, a negative outcome from frozen section analysis was less reliable than pre-operative imaging or surgical judgement.

#### 3.1.2. Laparoscopy

Staging laparoscopy’s (SL) role in the staging of PDAC is still uncertain. The procedure aims to reduce the frequency of unnecessary laparotomies in patients who, despite traditional pre-operative testing, appear to have resectable PDAC but have distant intra-abdominal metastases or vascular invasion that would prevent curative resection.

A meta-analysis conducted to assess the value of utilising SL for PDAC identified 24 studies and showed that the sensitivity of determining resectability was between 44 and 100%, while the specificity was within the range of 64–93% [20]. The authors also found an association with elevated levels of carbohydrate antigen (CA) 19.9 (92.77 to 353.15 U/mL) and carcinoembryonic antigen (CEA) [64] (2.47 to 5.5 ng/mL) in predicting unresectable disease in PDAC. The selection of surrogate markers for determining the appropriateness of SL in patients with CT-defined resectable pancreatic cancer remains a topic of ongoing research. Current evidence suggests that the most reliable surrogate markers for predicting resectability are CA 19.9 and tumour size. Although some studies have proposed the potential utility of tumour location (specifically, the body and tail of the pancreas), CEA levels [64], and clinical symptoms such as weight loss and jaundice, the existing evidence is insufficient to warrant their inclusion in a selection algorithm.

Fong et al. assessed the contemporary utility of staging laparoscopy for pancreatic adenocarcinoma by analysing data from 1001 patients who underwent surgical intervention for PDAC. The analysis showed that staging laparoscopy prevented unnecessary laparotomy for 44.4% of patients in the first period (2001–2008) and 24% of patients in the second period (2009–2015) [65]. The authors identified factors predictive of laparoscopically detectable metastatic disease, which were male gender, pre-operative resectability (borderline resectable and locally advanced), elevated CA 19.9 levels, absence of neoadjuvant chemotherapy, and pancreatic body or tail tumours. The researchers developed a scoring index based on these factors, which could predict 76.1% of radiographically occult metastatic disease, allowing for the selection of high-risk patients for laparoscopic biopsy and potentially avoiding unnecessary laparotomy. The study highlights the continued relevance of SL in the contemporary setting, despite improvements in imaging technologies and the use of neoadjuvant chemotherapy.

Overall, the use of SL in PDAC patients is still debatable, as several studies have challenged its efficacy. To better understand when and how SL should be used, more research is needed, including randomised controlled trials to quantify its effectiveness.

### 3.2. Emerging Techniques

#### 3.2.1. Lymph Node Mapping (LNM)

PDAC has a high incidence of perineural and lymphatic invasion and direct invasion of adjacent organs and structures that coincides with an increased morbidity when radical surgery is undertaken [66]. Metastasis in PDAC typically follows a haematogenous route to distant organs, including the liver, lungs, peritoneum, and bones, with most PDAC cases progressing in a skip metastasis pattern [67]. While the exact mechanism behind this form of metastasis is largely elusive, research suggests that skip metastases may be linked to tumour heterogeneity. Lymphatic mapping appears to be a necessary tool for detecting complex lymphatic systems in PDAC to plan an optimal surgical resection extent [68].

LNM for PDAC is a complex procedure that requires an experienced clinician for accurate intra-operative localisation. A radioactive substance or dye, such as methylene blue, is centrally injected into the tumour. Any LNs that become noticeable are subsequently removed for biopsy [69].

The sensitivity and specificity of LNM in PDAC vary depending on tumour size, the extent of regional LN involvement, and the surgeon’s skills. One study examined 13 patients with locally advanced PDAC (stage T3 or T4) with an average tumour size of 5 cm, where a 1-mL injection of blue dye (Guerbet) was administered into the tumour’s core to identify the sentinel node during surgery [68]. Only 5 patients (38.4%) were diagnosed using LNM, with the most common sentinel node located in the proximal splenic artery [68]. However, the investigation was unable to identify any patterns of lymphatic flow. A further study of 20 patients undergoing pancreaticoduodenectomy utilised indocyanine green (ICG) and found the lymphatic pathway reaching the para-aortic region in 85% of patients, with the following routes also identified: the anterior or posterior pancreaticoduodenal arcade, the superior mesenteric artery and vein, the colic artery, and the para-aortic region [70].

Few studies have shown the efficacy of LNM in PDAC, partly due to the mapping challenges that arise from lymphatic channels being involved in the malignant process [69]. These studies have further determined that the process of surgical sentinel LN detection in detecting metastases is expensive relative to the accuracy of results obtained, therefore favouring traditional methods [69,71].

#### 3.2.2. Intra-Operative Ultrasound (IOUS)

**Figure 1:**
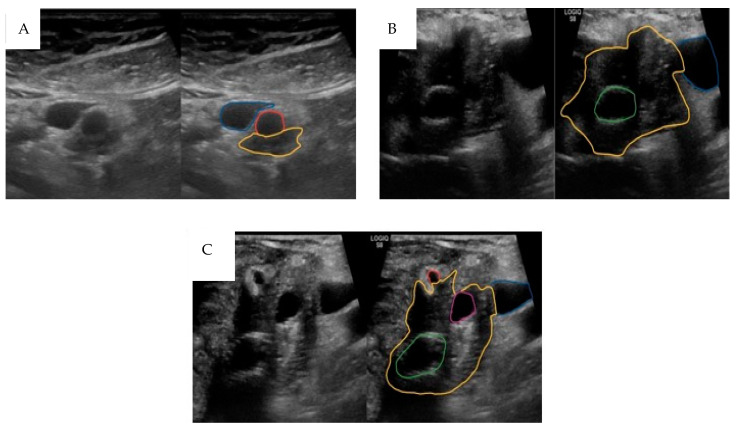
Representative non-annotated and annotated clinical images of ultrasound imaging of pancreatic ductal adenocarcinoma (PDAC). (**A**) Preoperative transabdominal ultrasound of pancreatic tumour extension (yellow) posterior to the 90–180 degrees affected superior mesenteric artery (unaffected wall marked red) and close to the superior mesenteric vein (blue, unaffected). (**B**) Intra-operative ultrasound of PDAC (yellow), with common bile duct stent (green) and <90 degrees affected superior mesenteric vein (unaffected wall in blue). (**C**) Intra-operative ultrasound of PDAC (yellow), with common bile duct stent (green) and 90 degrees affected superior mesenteric vein (unaffected wall in blue), 90 degrees affected gastroduodenal artery (unaffected wall in red), and dilated pancreatic duct (in purple). Data are original and unpublished.

Intra-operative ultrasound (IOUS) has been used to increase the accuracy of PDAC diagnosis and surgical assessment (Figure 1). This technique is often used to locate and identify tumours, evaluate their size and shape, and monitor progress of surgery [72].

IOUS has proven to be a valuable tool in managing pancreatic cancer [73]. A study by Cwik et al., which spanned over 10 years, involved 145 patients with pancreatic diseases, including confirmed and suspected malignancies. IOUS provided crucial insights into tumour staging, operability assessment, and identification of metastases, aiding in surgical decision-making with a diagnostic accuracy and sensitivity of 89.7% and 93%, respectively, surpassing other traditional imaging modalities. The technique allows for direct visualisation of the tumour mass, evaluation of its echostructure, and assessment of its borders and infiltration into adjacent structures. Additionally, IOUS enables the identification and imaging of LN involvement and metastases, particularly in the liver. This information is essential in planning the appropriate surgical approach, whether it involves tumour resection or palliation.

The diagnostic value of IOUS has been assessed in a recent prospective multicentre study that included patients (n = 85) undergoing surgical exploration after neoadjuvant chemotherapy for PDAC with vascular involvement. IOUS downstaged the resectability status in 38% of patients and enabled pancreatic resection, suggesting that the use of IOUS results in a significant change in resectability status [74]. Another study involving 1255 PDAC cases revealed a sensitivity of 76% and an NPV of 88% in assessing unresectable disease in PDAC using IOUS [44]. Machi et al. [75] found IOUS helpful in 73% of hepatobiliary and pancreatic procedures based on 1300 operations. This study revealed that IOUS offers valuable insights into many malignant and benign conditions, ranging from inflammatory to metastatic lesions. The analysis produced a sensitivity of 94.1%, a specificity of 86.4%, and an overall diagnostic accuracy of 89.7% when employing IOUS to diagnose PDAC.

This technology has many advantages that outweigh its disadvantages, including safety, speed, better imaging, and the ability to guide procedures. The drawbacks of this modality include the equipment required, a prolonged period to gain proficiency, and interference with visual capabilities if there is a significant amount of fat surrounding the pancreas [73].

#### 3.2.3. Intra-Operative Fluorescence-Guided Surgery (FGS): Diagnosis and Staging

Intra-operative fluorescence-guided surgery (FGS) utilises the principles of fluorescence imaging to enhance the visualisation of tumours during surgical procedures. By exploiting the unique properties of fluorescent dyes or probes, FGS provides high contrast and sensitivity, enabling macroscopic identification of cancerous tissue and delineating tumour margins with improved precision. FGS offers several advantages, including low cost, ease of use, the absence of ionising radiation, and high specificity. Optical-based FGS techniques are less expensive than traditional imaging methods, require less space, and seamlessly integrate into the surgical workflow [76], rendering them reducible to clinical practice.

FGS can potentially revolutionise the diagnosis of PDAC by providing real-time visualisation of tumour tissue during surgical resection. By utilising fluorescent probes specifically designed to target PDAC markers [77], FGS can enhance the accuracy of tumour localisation and the delineation of tumour margins. This precise identification of cancerous tissue can facilitate complete tumour resection and minimise the presence of residual tumour cells, which are strongly associated with tumour recurrence and poor survival outcomes. It also offers the advantage of reducing damage to critical structures and improving surgical decision-making. With ongoing advancements in the development of fluorescent probes and their translation into clinical practice, FGS holds promise as a valuable tool for early PDAC detection and improved treatment outcomes. 

**Figure 2:**
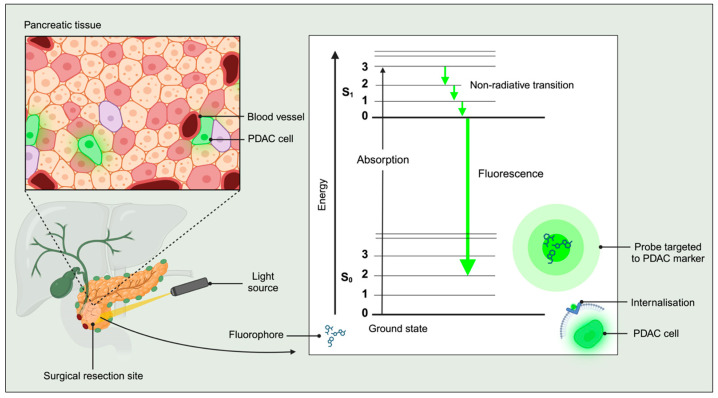
Excitation of a fluorophore for intra-operative fluorescence-guided surgery of PDAC. Fluorescent molecules (fluorophores) are taken up by PDAC cells due to specific cancer cell targeting. Light is delivered to the site of surgical resection and absorbed by the fluorophores, which adopt a higher energy excited singlet state (S_1_). The singlet state electrons then revert to their lower-energy ground state (S_0_), emitting photons of a lower energy (fluorescence) than the photons that had been absorbed by the fluorophore. This process, from absorption to emission, occurs within nanoseconds and is cyclical, meaning that as long as light is shone on the fluorophore-replete tissue, the tissue will emit fluorescence. PDAC cells therefore appear fluorescent, which enables the visualisation of cancerous cells and tissue during intra-operative fluorescence guided surgery. The figure was created on Biorender.com. Abbreviations: PDAC, pancreatic ductal adenocarcinoma.

When a fluorophore is excited by a light source, it will absorb the radiant energy (photons), elevating electrons to an energy level more significant than their ground state, the so-called first excited state. As the excited-state electrons return to the ground state, they release energy in the form of a photon whose wavelength is longer than the one initially absorbed. This is known as fluorescence, which always occurs in the visible range of the electromagnetic spectrum and is therefore perceptible by the human eye [76] (Figure 2).

Fluorescent labelling is helpful for laparoscopic surgery because it works with integrated systems that emit (light source) and detect (camera) specific wavelengths of light. The images produced by the fluorescent light can be superimposed onto standard white light images to avoid distorting the view of the surrounding anatomy. It can be challenging to discern fluorescent photos due to the autofluorescence emitted by molecules in non-malignant cells, such as mitochondrial proteins. Near-infrared (NIR) light (emitted wavelengths in the 650–900-nm range) is ideal for FGS because auto-fluorescence is significantly reduced and it allows for deeper tissue penetration. Accurate demarcation of the tumour in PDAC is critical for performing complete surgical resections, emphasising the importance of extra tools to compensate for the loss of tactile feedback in the age of minimally invasive surgery [78].

To improve the visibility of the original tumour, metastasised LNs, and distant metastases, effective and safe tumour-specific NIR fluorescence tracers have been developed and employed over the past few years for PDAC imaging. These fluorophores are covalently linked to antibodies that specifically cross-react with cognate antigens expressed in the region of interest, allowing for FGS to be tumour-specific. This will enable surgeons to identify the areas of interest quickly and accurately without the need for invasive biopsies [76]. Several fluorophores have been studied for FGS in PDAC, including 5-aminolevulinic acid (5-ALA, which is converted to the fluorescent protoporphyrin IX (PpIX)), ICG, and fluorescein. 5-ALA is the most widely studied fluorogenic agent for FGS in PDAC (Table 1) [79,80]. PpIX can be visualised in malignant cells using a blue light source for excitation [81]. FGS allows surgeons to identify cells that may have been missed through traditional diagnostic procedures and guide surgical resection to detect metastases to the liver and peritoneum, enabling a more precise treatment [82].

5-ALA is a prodrug and is naturally present in most cells. It is metabolised via the heme biosynthesis pathway to PpIX. Upon blue light excitation, PpIX emits red fluorescence, which is conducive to laparoscopic surgery. In normal cells, 5-ALA synthesis is regulated by negative feedback due to high intracellular concentrations of heme. However, this negative feedback system is overridden when exogenous 5-ALA is administered. Alterations in the rate of activity of porphobilinogen deaminase (PBGD) and ferrochelatase enzymes are a key factor in determining PpIX selective accumulation in tumour cells. Intracellular PpIX returns to normal levels 48 h after administration of 5-ALA, owing to active outward transport via the membrane transporter ABCG2(ATP-binding cassette super-family G member 2), which provides an ample time window for fluorescence-guided surgery. 

The application of FGS is a relatively new concept in clinical trials, mainly in phases I/II. Still, it is quickly gaining interest among medical professionals due to its potential for providing real-time information about tissues during surgery [83] whilst not requiring notable expertise to interpret visual output or dedicated instrumentation. FGS can be used with other imaging techniques, such as CT and MRI, to provide an even more comprehensive view of the organ being operated on.

Although a very promising instrument, FGS does come with caveats that are relevant to PDAC staging, defining resection margins, and the visualisation of residual tumour cells as well as metastatic sites. First and foremost, tumour cells can only be fluorescently tagged if the fluorophore can physically interact with its intended target. For that to occur, tumour (micro)circulation is required in combination with extensive diffusion, which is sometimes lacking in the tumour periphery, residual tumour cells outside the FGS-defined resection margins, small, non-vascularised tumors, and micrometastases. The fluorescent demarcation of a tissue bulk does not per se represent a clear-cut border between malignant and benign tissue, and resection margins should still be set a distance from the dark-fluorescence interface. Other tools, such as those described above (e.g., IOUS), will provide a more constructive picture of the overall pathological environment. With respect to micrometastases, these clumps of tumour cells are often sufficiently oxygenated and therefore are not vascularized. Fluorescence guidance can therefore not be applied in a strict sense, and, although exceptions apply as described in the next section, the absence of fluorescence can by no means be interpreted as a metastasis-free state.

##### Fluorescence for Tumour Visualisation

A recent phase I clinical study investigated the efficacy of intravenously administered panitumumab-IRDye800CW, a fluorescently labelled anti-epidermal growth factor receptor (EGFR) antibody, in the surgical management of PDAC [84]. Sixteen patients were enrolled in the study, who were injected with doses of 25 mg, 50 mg, or 75 mg before surgery. The authors reported that intra-operative fluorescence imaging demonstrated enhanced visualisation of primary tumours compared to surrounding normal tissue in all three dosing cohorts, while the highest tumour-to-background ratio (TBR) was observed in the 50 mg dose cohort. Additionally, fluorescence imaging successfully identified metastatic LNs and small peritoneal metastases (≤2 mm) that were not readily detectable using conventional imaging techniques, accounting for a sensitivity and specificity of 90.3% and 74.5%, respectively.

A further phase I clinical trial utilised SGM-101, a fluorescently labelled anti-CEA antibody [64], in 12 patients undergoing surgical exploration for PDAC [85]. Fluorescence imaging using SGM-101 (10 mg dose, 96 h post-administration) was feasible and allowed the detection of primary tumours and liver- and peritoneal metastases. However, the tumour-to-background ratios (TBRs) were more modest compared to other tumour types, potentially due to the histopathology of PDAC that is characterised by sparse fluorescence patterns in the presence of desmoplastic stroma. Intra-operative false-negativity cases were attributed to overlying tissue and blood, limiting the detection of deeply seated or (optically) covered tumours. To enhance detection, the study suggested combining fluorescence with other imaging modalities, such as radionuclides or photo-acoustic imaging. CEA was confirmed as a suitable target for fluorescence imaging in PDAC.

Another prospective case study by Katada et al. involving 133 consecutive pancreatic cancer patients with no evident hepatic metastases on pre-operative imaging assessed the efficacy of FGS [86]. Intra-operative fluorescence imaging using ICG and a near-infrared camera system was employed to detect hepatic micrometastases during surgery, and pre-operative imaging was retrospectively reviewed. The findings revealed that fluorescence imaging proved to be a highly sensitive method for detecting hepatic micrometastases in real time, with 15% of patients showing histologically confirmed micrometastases. These micrometastases were characterised by portal thromboemboli in the intrahepatic portal triad that invaded extravenous structures, causing desmoplastic reactions and local biliary obstruction. Notably, fluorescence imaging also detected altered local circulation and bile stasis at the portal triad, which were not discernible in conventional radiological imaging.

It should be noted that ICG is avidly taken up by hepatocytes via multiple basolateral transporters and excreted into the biliary system without biotransformation, which can last up to 20 h after an intravenous bolus challenge [87]. Accordingly, ICG is used to assess liver function in the clinical setting [88]. In this respect, employing ICG for FGS must be preceded by a sufficiently long hepatic clearance period to allow complete removal of background fluorescence from normal liver parenchyma while retaining the fluorophore in the metastatic nodule, which lacks the ICG efflux transporter and/or the clearance infrastructure, as was done by Katada et al. (24 h clearance period) [86].

The studies nevertheless demonstrate that fluorescence imaging, with its ability to identify subtle alterations in liver tissue during surgery, plays a crucial role in visualising hepatic micrometastases from pancreatic cancer. This technique offers an early detection tool to guide surgeons in accurately identifying and resecting micrometastases, potentially impacting treatment planning and patient outcomes. The findings highlight the clinical significance of fluorescence imaging as a valuable intra-operative tool for tumour visualisation that could improve the management of patients with pancreatic cancer and suspected hepatic metastases. However, further research with larger patient cohorts is warranted to confirm and validate these results.

A summary of all clinical studies that examined the efficacy of FGS in the management of pancreatic cancer is presented in Table 1.

##### Fluorescence Lymph Node Imaging

Several studies have been conducted to investigate the use of fluorescence for LN detection in PDAC. It has been proposed that numerous tumour-specific surface antigens, including integrin αvβ6, CEA [64], EGFR, and urokinase plasminogen activator receptor (uPAR), have the potential to be employed primarily for detecting PDAC [89].

In the first-in-man study, Tummers et al. [90] studied the feasibility of applying molecularly guided fluorescent imaging technology to identify LNs with metastatic PDAC in patients undergoing surgical resection, using a combination of CT and NIR fluorescence imaging in a dose-escalation prospective study. Two doses of an anti-EGFR antibody, Cetuximab-IRDye800CW (50 mg and 100 mg), were tested and 144 dissected LNs from 7 patients were examined ex vivo using macroscopic and microscopic fluorescent imaging. The results of the imaging were then compared with histopathology findings. The low-dose cohort showed highly reliable detection of metastatic LNs when using fluorescence, with 100% sensitivity and 78% specificity on macroscopic observation and 91% sensitivity and 66% specificity on microscopic observation. Encouragingly, this approach demonstrated the ability to detect tumour foci of less than 5 mm, corresponding to a sensitivity of 88%. The evidence for fluorescence detection of nodal metastases, which was not previously seen with CT imaging, was shown in these studies, even if they were from ex vivo imaging.

This research delivers evidence that molecularly targeted fluorescence imaging can identify tumour-positive peripancreatic LNs ex vivo with considerable precision even when dealing with microscopically challenging cases. Consequently, if applied in vivo, this method offers more significant visibility for surgeons during an operation. It could result in more accurate and focused LN dissection. For fluorescence imaging to be deployed in broader surgical applications, phase III multicentre studies must be undertaken.

#### 3.2.4. Optical Coherence Tomography (OCT)

**Figure 3:**
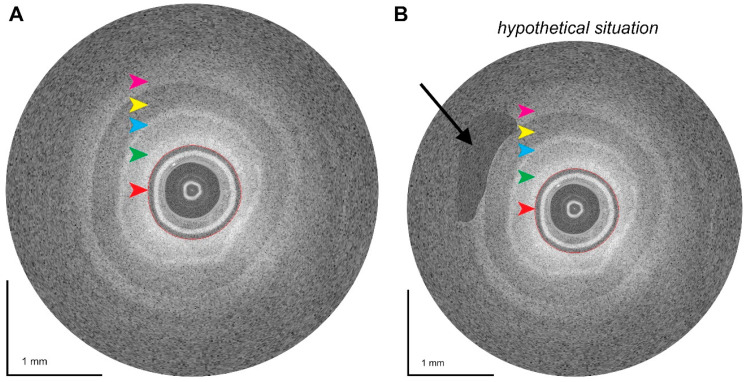
The potential utility of intravascular optical coherence tomography (OCT) to determine vascular involvement of PDAC, which is a key variable that dictates resectability. (**A**) Ex vivo intravascular OCT image of a normal gastroduodenal artery wall after a Whipple resection in a patient with pancreatic ductal adenocarcinoma. The red arrowhead and red circle delineate the OCT catheter. Four recognisable layers are visualised, which are hypothesised to represent the intima (green arrowhead), the internal elastic lamina (blue arrowhead), the media (yellow arrowhead), and the adventitia (magenta arrowhead). (**B**) Illustration of a hypothetical scenario where intravascular OCT is used in a blood vessel with tumour involvement at the level of the arterial wall (black arrow). Instead of the homogeneous wall layers shown in the normal gastroduodenal artery wall (**A**), panel (**B**) shows heterogeneous back-scattering due to the presence of compositionally distinct tumour tissue. It should be noted that intravascular OCT for PDAC constitutes a novel approach that currently is being validated. Data are original and unpublished.

The early stages of optical coherence tomography (OCT) were established by D. Huang and colleagues in the 1990s [91]. OCT employs infrared light waves to generate fine-grained cross-sectional images of tissue by measuring the reflected or backscattered intensity of light from microstructures within tissue as a function of depth [92]. It is comparable to ultrasound, but instead of sound, it employs light. OCT can be used to quickly identify aberrant tissue changes, malignant cells, and physiological events, which harnesses the potential to help physicians decide on the best course of action (Figure 3). OCT is gaining popularity for cancer screening and diagnosis and has been used to image the eyes, heart, oesophagus, and other organs [93]. The advent of artificial intelligence will likely enhance image analysis capabilities and diagnostic accuracy of OCT and expedite its adoption (Figure 3).

**Table 1:** Features of clinical trials using FGS for intra-operative staging of PDAC.

Study Type	Number of Patients	Fluorescent Agent	Targeted	Fluorescence Emission Wavelength	Dose	Intra-Op Detection (Tumour, Lymph Nodes, Micrometastasis)	Complications/Limitations	Reference
Prospective	49	Indocyanine green	NO	600 to 900 nm	2.5 mg/mL	Improved visualisation of tumour and detection of micrometastasis	Background fluorescence was significantly greater than that of the tumour False-positive fluorescence was not true false-positives as in 60% of these patients hepatic recurrence occurred Inadequate deep tissue penetration of near-infrared light	[94]
Prospective	133	Indocyanine green	NO	600 to 900 nm	2.5 mg/mL	Increased detection of unsuspected liver metastasis from PDAC	No complications reported	[86]
Case report	1	Methylene blue	NO	700 nm	1.0 mg/kg	LN detection and potential tumour visualisation	No complications reported	[95]
Phase I dose-escalation	16	Panitumumab-IRDye800CW	Anti-EGFR antibody	800 nm	25, 50, or 75 mg	Emphasised surgical margins, LN metastasis and distant metastasis	Hypertension in 1 patient Post-surgical presyncope in 1 patient Prolongated QTc after infusion in 1 patient Vomiting in 1 patient	[84]
Prospective protocol	40	Indocyanine green	NO	600 to 900 nm	0.5 mg/kg	Improves visualisation in PDAC and peritoneal metastases	No complications reported	[96]
Clinical trial	12	SGM-101	Anti-CEA antibody	700 nm	5, 7.5, or 10 mg	Safe and viable for intra-operative identification of both primary PDAC and liver and peritoneal metastases	False-negatives and false-positives witnessed No detection of deeply seated tumours	[85]
Phase I dose-escalation	7	Cetuximab-IRDye800	Anti-EGFR antibody	800 nm	50 mg or 100 mg	Improved visualisation of LN metastases. Effectively detected tumours exhibiting a notably elevated average fluorescence intensity within the tumour compared to the surrounding normal pancreatic tissue and cases of pancreatitis	Increased false-positives fluorescent LNs at 100 mg Small sample size In the case of PDAC, relying solely on fluorescence as a single modality for imaging, with an achievable depth of 1 cm, falls short of effectively capturing the spatial characteristics of the tumour	[97]

Abbreviations: EGFR, endothelial growth factor receptor; CEA, carcinoembryonic antigen; LN, lymph node.

In terms of PDAC, an old study involving 29 patients who had undergone surgery for suspected pancreatic cancer utilised a training set of 100 OCT images that were assessed by two pathologists [98]. Fresh tissue samples were collected from 17 patients with (pre)malignant pancreatic lesions, and formalin-fixed paraffin-embedded (FFPE) samples were collected from 12 patients with benign and malignant pancreatic neoplasms. The pathologists scored the full field (FF)-OCT images as malignant or benign, and the results were compared to those obtained with standard haematoxylin and eosin (H&E) slides. Both pathologists’ overall combined test characteristics showed a sensitivity of 72%, specificity of 74%, positive predictive value of 69%, NPV of 79%, and overall accuracy of 73%. At least one pathologist interpreted 97% of the FF-OCT images as tumours in the subset of PDAC patients. Pathologies such as atrophy, fibrosis, and neuroendocrine tumours were incorrectly scored. The study concluded that using architectural features, FF-OCT could distinguish normal pancreatic tissue from pathologic tissue in processed and non-processed specimens.

A systematic review of the effectiveness of OCT in PDAC found that, when testing ten patients, OCT was associated with a specificity and sensitivity rate of 88.9% and 78.6%, respectively [99]. OCT recognised a definite, different pattern in 82.9% of tumour-free and 97.6% of tumour-involved specimens. The sensitivity and specificity for discrimination between adenocarcinoma and normal tissue were 78.6% and 88.9%, respectively. Inflammatory and dysplastic changes in the main pancreatic duct showed an OCT pattern similar to that of the normal tissue in 53.3% of images, reflecting the value of this technique in regard to discrimination between different pathologies. Corroboratively, the study assessed the intra- and interobserver reproducibility of real-time OCT images. Overall, intra-observer reproducibility ranged from 85.1% to 100%, and inter-observer reproducibility ranged from 69.9% to 100% and from 89.7% to 100% for tumour-free and tumour-involved segments, respectively. The study concluded that OCT could be beneficial for identifying early ductal epithelial changes and for differential diagnosis between inflammatory and neoplastic lesions.

Before integrating the FF-OCT device into the clinical practice of pancreatic surgery, it is imperative to enhance its development further whilst also gaining additional expertise in assessing FF-OCT images of the pancreas, by discerning benign from malignant diseases [98]. A potential aim to improve the diagnostic accuracy of PDAC using OCT would be to study the arrangements of collagen fibres in a range of benign and malignant conditions, as these tend to differ drastically, which could further allow accurate diagnosis based on histological features.

#### 3.2.5. Radioimmunoguided Surgery (RIGS)

RIGS utilises a radiation-detection device within the operating theatre to identify a radiolabelled antibody or derivative administered intravenously before the surgery. A phase I/II study involving ten patients diagnosed with PDAC who were intravenously injected with 1 mg of CC49 monoclonal antibody (targets TAG-72, a type of mucin associated with adenocarcinomas) that was radiolabelled with 2 mCi iodine 125 found an additional four visceral sites of disease using RIGS as opposed to exploratory laparotomy [100]. More importantly, the exploratory laparotomy revealed the presence of 6 areas of lymphatic disease, which was drastically lower than the total of 44 regions of lymphatic disease found with the RIGS technique (*p* < 0.001). This new method of exploring the spread of disease among LNs provides far superior results compared to traditional approaches.

However, the literature on PDAC could be more extensive [101]. Due to the variable sensitivity and difficulties in handling and disposing of radioactive material, the technique has become relatively obsolete [102]. Further drawbacks identified revealed a profusion of false-positive tissue detections, which is its biggest flaw, and with it increasing procedural times, making the putative advantages of this technique unsustainable in terms of logistics and cost [103].

#### 3.2.6. Ultrasound Elastography (UEG)

UEG enables the virtual palpation of the pancreas, which estimates its tissue stiffness. EUG is based on plotting the strain of a tissue arising from an external compression force, like the one produced by an ultrasound transducer. The greater the music, the softer the tissue, while lesser pressure corresponds to more inflexible tissues. UEG can be utilised for the further distinction of pancreatic healthy tissues and masses by observing the tissue strain from external compression [104]. A previous study involving 44 patients attested to the utility of endoscopic UEG (E-UEG) in diagnosing vascular invasion in PDAC, particularly in instances where vascular invasion could not be accurately diagnosed by CT [105]. In determining the presence of vascular invasion in PDAC, the sensitivity and specificity of E-UEG were 92 and 90%, respectively, compared to 73% and 60% for dynamic CT [105].

Furthermore, Inoue et al. [106] demonstrated the differences in elasticity between benign and malignant liver tumours in 56 lesions. This study corroborated earlier findings by Kato et al. [107], who employed UEG to intra-operatively examine 6 PDAC lesions and demonstrate the safety of the approach. Compared to findings with intra-operative greyscale US alone, elastographic imaging was shown to be effective in evaluating the characteristics of the tumour, such as the absence of vascular involvement, without surgical complications.

UEG can potentially be used to diagnose and stage PDAC lesions. Nevertheless, a significant constraint of UEG evaluation is its limited ability to accurately discern the precise characteristics of pancreatic masses and therefore EUS-FNA surpasses this modality in concluding an accurate diagnosis.

#### 3.2.7. Overview Intra-Operative PDAC Imaging 

The techniques that have been addressed in this review are summarised in terms of the most clinically important parameters in Table 2.

## 4. Conclusions and Prospects

Currently, we still do not have a robust technique or combination of modalities that provides accurate intra-operative staging of PDAC, particularly the localisation of metastatic LNs and extra-pancreatic pathology. Several intra-operative techniques have been investigated for these purposes, and tumour-specific fluorescence-guided surgery appears to be a promising tool in diagnosing and treating PDAC. This is tackling a critical issue in PDAC where early recurrence occurs despite supposed R0 resections. Although FGS harnesses a lot of potential, further research is needed to fully understand the potential applications and benefits of fluorescence imaging for pancreatic cancer surgery. Ultimately, tumour-specific FGS combined with other diagnostic and therapeutic modalities could improve outcomes for patients with PDAC. Given the rapid advancements in clinical artificial intelligence (AI) technologies such as radiomics, pathomics, genomics, and surgomics, new opportunities are likely to emerge, transforming clinical pathways in pancreatic cancer surgery [108]. The successful integration, in the future, of AI-driven machine learning algorithms and FGS in operating theatres holds the potential for a paradigm shift. These innovations could assist surgeons in real-time by precisely analysing the extent of the tumour as well as pertinent anatomical features during surgery.

## Figures and Tables

**Table 2:** Intra-operative techniques for the staging of PDAC.

Technique	Detection Modality	Tumour Specific	LN Specific	Advantages	Disadvantages
Frozen section	Histological assessment	Yes	Yes	Rapid histological assessment of tumour cells presence in tissue samples	Operator dependent, high false-negative rate, anatomical accessibility limitation
SL	Direct visualisation	No	No	Minimally invasive	Non-specific visualisation of abdominal solid organs and peritoneum, operator-dependent, inherent laparoscopy complications
LNM	Dye/fluorescence	No	Yes	Minimally invasive, with fewer complications than lymphadenectomy	More invasive procedure than other diagnostic tests, difficult data interpretation, time consuming
IOUS	Ultrasound transducer	No	No	Real-time visualisation, guides surgical resection margins, performed in a relatively short amount of time	Expertise needed for the ultrasound operator. Need better visualisation of the target area. Limited by excess fat in the area, determined by the size of the ultrasound probe
FGS	Fluorescence	Yes	Yes	Provides both static and dynamic images, molecular level detail. No radiation exposure	Accuracy dependent on the agent used
OCT	Optical probe and interferometer	No	No	High-resolution, non-invasive	Penetration depth is limited, expensive, expertise required
RIGS	Hand-held gamma probe	Yes	Yes	Sensitive, specific, safe	Radioactive material, handling and disposal constraints, time consuming
UEG	Ultrasound transducer	No	No	Non-invasive, inexpensive, safe	Steep learning curve, low specificity

Abbreviations: LN, lymph node; SL, staging laparoscopy; LNM, lymph node mapping; IOUS, intra-operative ultrasound; FGS, fluorescence guided surgery; OCT, optical coherence tomography; RIGS, radioimmunoguided surgery; UEG, ultrasound elastography.
